# Prevalence of Diagnosed Essential Tremor in the United States: An Administrative Claims-Based Study

**DOI:** 10.5334/tohm.1060

**Published:** 2025-10-21

**Authors:** Junji Lin, Rajesh Pahwa, Elan D. Louis, Ragy Saad, Kelly E. Lyons, Michael Markowitz, Liza R. Gibbs, Aisara Chansakul, John Kroner, Douglas S. Fuller, Weiyi Ni, Arthur Sillah, Michelle Baladi, Luigi M. Barbato, Sanket Shah

**Affiliations:** 1 Jazz Pharmaceuticals, Palo Alto, CA, USA; 2 University of Kansas Medical Center, Kansas City, KS, USA; 3 University of Texas Southwestern Medical Center, Dallas, TX, USA; 4 Former employee of Jazz Pharmaceuticals; 5 Aetion Inc, New York, NY, USA; 6 Jazz Pharmaceuticals, Philadelphia, PA, USA

**Keywords:** essential tremor, prevalence, real-world evidence, real-world data, administrative claims

## Abstract

**Background:**

Essential tremor (ET) is one of the most common movement disorders. Previous estimates of ET prevalence vary due to multiple factors. This study estimated the prevalence of diagnosed ET.

**Methods:**

The prevalence of diagnosed ET was estimated among adults with continuous enrollment in 2022 and ≥1 additional year of prior baseline enrollment in the Merative™ MarketScan^®^ Research Databases, a US administrative claims database. ET was defined as ≥2 ET claims within 12 months of one another during the 2016–2022 study period. The proportion of patients receiving treatment was defined as having a claim for possible medication for ET in 2022 among those with diagnosed ET who had an additional 6 months of follow-up following the first ET diagnosis claim. Age-standardized estimates of the number of US adults with diagnosed ET were calculated using 2024 US population census projections.

**Results:**

The prevalence of diagnosed ET was 0.28% before age standardization, ranging from 0.06% (18–40 years) to 1.61% (≥75 years); 74% of patients overall received possible treatment. After standardization, prevalence was 0.42%; in 2024, 1.1 million US adults were estimated to have diagnosed ET. Estimates of the prevalence of diagnosed ET in the US were susceptible to the choice of case definition, nearly doubling (ie, 2.1 million US adults) with a more sensitive definition.

**Discussion:**

ET affects a substantial proportion of the US adult population. Selecting appropriate case definitions and using methods such as standardization are critical for estimating valid and generalizable chronic condition prevalences with real-world data.

**Highlights:**

This study found that 1.1 million US adults were estimated to have been diagnosed with essential tremor, with the sensitivity analyses yielding additional estimates. Estimating reliable and generalizable prevalences of diagnosed chronic conditions requires selection of appropriate case definitions and standardization to the general population.

## 1. Introduction

Essential tremor (ET), a progressive neurological disease characterized by an action tremor, is the most common tremor disorder among adults [1,2,3,4,5,6]. The tremor can affect activities of daily living [4,5]. Currently available treatments for ET aim to improve tremor, and may be utilized by those whose tremor results in functional disability [3,4,5,7]. Discontinuation of therapy can be common due to side effects and limited effectiveness of currently available treatments [2,4,7].

First-line treatments for ET include propranolol, a beta blocker that is the only approved medical treatment for ET in the United States (US), and off-label use of primidone, an anticonvulsant [3,8]. A number of off-label medications may be recommended as second- and third-line treatment options [3]. Approved neurosurgical procedures include focused ultrasound and deep brain stimulation and are recommended as options for patients with disabling ET that is refractory to pharmacological therapy [3,4,9]. A literature review of real-world use of pharmacotherapies for ET found that up to 81% and 55% of patients with ET used propranolol and primidone, respectively, but the proportion who discontinued the medications ranged from 10% to 70% [10].

Meta-analyses of multinational studies have estimated the pooled overall prevalence of ET to range from 0.32% to 1.33% worldwide, with an estimated prevalence of 2.2% in the US via extrapolation of data from populations in Turkey, Israel, and Spain [11,12,13]. However, estimates of the prevalence of ET may differ due to a variety of factors including a high rate of misdiagnosis and evolving diagnostic criteria [14,15,16,17]; differences in study sample age distribution given the increasing prevalence of ET observed with increasing age; and heterogeneity in case definitions and data sources used [11,13,18,19]. A previous study used administrative claims data to estimate the burden of ET in the US, but it was not primarily focused on prevalence estimation [20].

Population-based studies have focused on the overall prevalence of ET, including subclinical or untreated cases, rather than the prevalence of cases with diagnosed ET. Estimates of the proportion of cases in the population who seek a diagnosis and receive treatment are on the order of approximately 10% [21]. Furthermore, until the implementation of ICD-10 in 2016, there was no specific diagnosis code for ET [22,23]. As a result, claims-based analyses, although helpful in determining the prevalence or incidence of related conditions, would have been constrained by the high likelihood of misclassification in the case of ET [24,25]. Due to these factors, estimating the diagnosed ET population has proven to be a challenge in the past. Hence, this study aimed to: 1) estimate the prevalence of diagnosed ET among adults in the US using recent data from a real-world US claims data source among an observable population, including evaluation of robustness of estimates by varying the sensitivity of the ET-case definition; 2) assess treatment utilization among patients diagnosed with ET; and 3) apply prevalence estimates to current US population data to estimate the number of adults currently diagnosed with ET in the US and the number of these patients receiving possible treatment for ET.

## 2. Methods

### 2.1. Design and data source

This study was a retrospective cohort study using Merative™ MarketScan^®^ Research Databases spanning January 1, 2016, through December 31, 2022 (the study period). MarketScan is a US administrative healthcare claims database that provides longitudinal, deidentified service-level data on US patients’ plan enrollment, demographics, healthcare utilization, inpatient and outpatient events, and pharmacy dispensing for billed care. Individuals with both commercial and public coverage are included, with these data including both the Commercial Claims and Encounters Database and the Medicare Supplemental and Coordination of Benefits Database. As a secondary analysis of deidentified data, this study did not require institutional review board approval.

### 2.2. Prevalence Denominator Cohort and Prevalent ET-Case definitions

To estimate the prevalence of diagnosed ET during the most recent calendar year of available data (2022), the Prevalence Denominator Cohort included individuals who met the following criteria: 1) Continuously enrolled with up to a 30-day gap allowed between January 1, 2021, and December 31, 2022 (continuous medical and pharmacy enrollment throughout 2022 and with at least one year of enrollment throughout 2021 to ensure baseline observability); and 2) At least 18 years of age on January 1, 2022, to restrict to a denominator population of adults in 2022.

In the primary analysis, individuals in this Prevalence Denominator Cohort were considered to be Prevalent ET Cases upon the second of two medical claims with a diagnosis code for ET (ICD-10-CM G25.0) in any position in the inpatient or outpatient setting during the 2016–2022 study period, where the second claim started at least 1 day and at most 365 days after the first claim (Primary ET-Case Definition). In the absence of available validated algorithms to identify ET in claims data, this definition was chosen for its consistency with other validated identification algorithms for neurological conditions [26,27]. For sensitivity analyses, two alternative case definitions of ET were additionally evaluated to assess the impact of using more sensitive definitions of ET: 1) ET defined as in the primary analysis, but allowing up to 730 days between claims (expanded from 365 days) and 2) ET defined as at least one medical claim with a diagnosis code for ET during the 2016–2022 study period (rather than at least two claims), as described in recent literature [20].

### 2.3. Prevalent ET-Case Denominator Cohort and possible treatment for ET definitions

To estimate the proportion of patients with prevalent diagnosed ET who received treatment, the Prevalent ET-Case Denominator Cohort included those who met the above Primary ET-Case definition and who additionally had at least six months of continuous enrollment (with a 30-day gap allowed) after meeting the Primary ET-Case Definition, to ensure that all patients had at least six months of observability following ET diagnosis during which to evaluate treatment utilization. The 30-day maximum gap ensures that all patients are continuously enrolled and is widely used in claims analyses [28,29].

Individuals in this Prevalent ET-Case Denominator Cohort were considered to have received possible treatments for ET during the prevalence year of interest (2022, the last calendar year of available data within the claims database) if they had a prescription claim in 2022 for any medication with evidence indicating potential efficacy for treatment of ET (**Supplemental Table 1**) [6,9,30,31] on or after meeting the Primary ET-Case Definition. Individuals in the Prevalent ET-Case Denominator Cohort who had a qualifying claim for treatment prior to but not during 2022 were not considered to have received 2022 treatment. Pharmacologic treatments were described overall and by medication class and generic name. Additionally, the proportion of patients with claims for non-pharmacological treatments including deep brain stimulation, radio-surgical gamma knife thalamotomy, and focused ultrasound was described.

**Figure 1:**
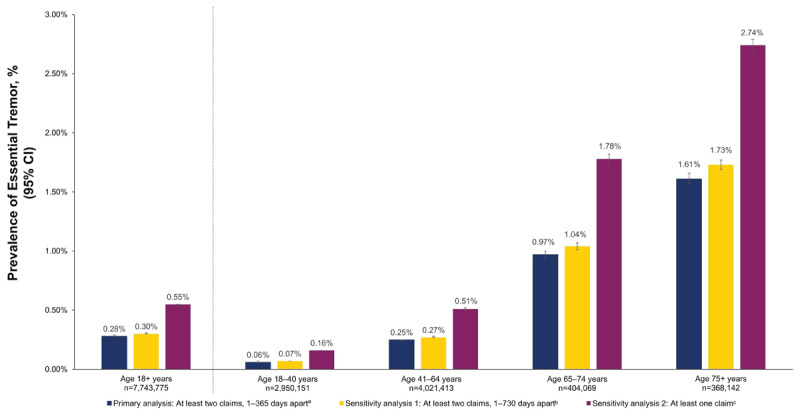
Estimated Prevalence of Diagnosed Essential Tremor by Age Group and Case Definition (2022 Data From MarketScan). ^a^Primary analysis: patients are considered to have diagnosed ET upon the second of at least two claims with a diagnosis of ET between 1 and 365 days apart during the study period. ^b^Sensitivity analysis 1: patients are considered to have diagnosed ET upon the second of at least two claims with a diagnosis of ET between 1 and 730 days apart during the study period. ^c^Sensitivity analysis 2: patients are considered to have diagnosed ET with a record of at least one ICD10 code, at any time during the study period. The study period starts on 1/1/2016 and extends through 12/31/2022; n represents the respective denominator in the MarketScan data.

### 2.4. Prevalence and proportion calculations

The prevalence of diagnosed ET was calculated by dividing the total number of Prevalent ET Cases by the total number of individuals in the Prevalence Denominator Cohort, overall and among the following age groups at the time of meeting the Primary ET-Case Definition: 18–40 years, 41–64 years, 65–74 years, and ≥75 years. The proportion of patients with diagnosed ET who received possible treatment for ET was calculated by dividing the total number of eligible patients receiving a presumptive pharmacologic treatment for ET by the total number of patients in the Prevalent ET-Case Denominator Cohort (as defined in the primary analysis), overall and by age group. For prevalence and treated proportions, 95% confidence intervals (CIs) were estimated using the binomial proportions distribution as follows: letting n be the number of patients and p the observed proportion of successes, if np(1-p) <5, the Clopper-Pearson method was used; otherwise, the Wald interval was used [32].

### 2.5. Age standardization to estimate the prevalence of diagnosed ET in the US

The number of adults in the US with diagnosed ET in 2024 within each age group was estimated by multiplying the age-group specific prevalence estimates by the corresponding US population according to 2024 estimates from the International Database of the U.S. Census Bureau International Programs Center [33]. Though prevalence was estimated as of 2022 data, age standardization was performed according to 2024 population estimates in order to estimate the 2024 prevalence of ET in the US. Given that there have been no significant changes in terms of the American Academy of Neurology diagnosis criteria and treatment guidelines for ET patients over the last two years, this study anticipates no differences in diagnosis prevalence between 2022 and 2024 [34]. The age-standardized estimate of the total number of adults in the US with diagnosed ET was calculated by summing the age-specific estimated number of adults with ET across all adult age groups. Age standardization was utilized to account for differences in the age distribution in the overall US population as compared with the study sample denominator population, given increasing prevalence of ET with increasing age [11,13,18,19]. Using age-specific estimates of the proportion of individuals with ET who receive possible treatment for ET, age-adjusted estimates of the number of individuals in the US with ET who receive treatment were additionally calculated.

## 3. Results

### 3.1. Prevalence of diagnosed ET in the study population

Among 7,743,775 adults meeting the study inclusion criteria for the Prevalence Denominator Cohort during the 2016–2022 study period, 38.1% were age 18–40 years at the start of the prevalence assessment year (2022), 51.9% were age 41–64 years, 5.2% were age 65–74 years, and 4.8% were age ≥75 years (Table 1). Using the Primary ET-Case Definition, which required at least two ET-related claims within one year during the study period, the overall prevalence (95% CI) of ET was 0.28% (0.28%, 0.29%) (Figure 1). Allowing up to two years between ET claims during the study period resulted in an overall prevalence (95% CI) of 0.30% (0.30%, 0.31%); expanding case criteria to require only one claim during the study period resulted in an overall prevalence (95% CI) of 0.55% (0.54%, 0.55%). Using the Primary ET-Case Definition requiring at least two ET-related claims within one year during the study period, the age-specific prevalence (95% CI) of ET numerically increased with age (ie, 0.06% [0.06%, 0.07%], 0.25% [0.24%, 0.25%], 0.97% [0.94%, 1.00%], and 1.61% [1.57%, 1.66%] for those aged 18–40, 41–64, 65–74, and ≥75 years, respectively). In each sensitivity analysis, the age-specific prevalence also numerically increased with age.

**Table 1:** Prevalence of diagnosed essential tremor (ET) in MarketScan in 2022, among adults overall and by age subgroup.


	OVERALL: AGE ≥18 YEARS *NOT AGE-STANDARDIZED*	AGE SUBGROUPS			

		AGE 18–40 YEARS	AGE 41–64 YEARS	AGE 65–74 YEARS	AGE 75+ YEARS

Individuals at-risk^a^; N (% of all adults at-risk)	7,743,775 (100.0)	2,950,151 (38.1)	4,021,413 (51.9)	404,069 (5.2)	368,142 (4.8)

**Primary analysis: At least two claims, 1–365 days apart** ^b^					

Patients with prevalent diagnosed ET; N	21,780	1,894	10,028	3,915	5,943

Prevalence of diagnosed ET; % (95% CI)	0.28 (0.28, 0.29)	0.06 (0.06, 0.07)	0.25 (0.24, 0.25)	0.97 (0.94, 1.00)	1.61 (1.57, 1.66)

Prevalence of diagnosed ET; per 1,000 patients (95% CI)	2.81 (2.78, 2.85)	0.64 (0.61, 0.67)	2.49 (2.44, 2.54)	9.69 (9.39, 9.99)	16.14 (15.74, 16.55)

**Sensitivity analysis 1: At least two claims, 1–730 days apart** ^c^					

Patients with prevalent diagnosed ET; N	23,576	2,079	10,926	4,214	6,357

Prevalence of diagnosed ET; % (95% CI)	0.30 (0.30, 0.31)	0.07 (0.07, 0.07)	0.27 (0.27, 0.28)	1.04 (1.01, 1.07)	1.73 (1.69, 1.77)

Prevalence of diagnosed ET; per 1,000 patients (95% CI)	3.04 (3.01, 3.08)	0.70 (0.67, 0.73)	2.72 (2.67, 2.77)	10.43 (10.12, 10.74)	17.27 (16.85, 17.69)

**Sensitivity analysis 2: At least one claim** ^d^					

Patients with prevalent diagnosed ET; N	42,439	4,696	20,455	7,203	10,085

Prevalence of diagnosed ET; % (95% CI)	0.55 (0.54, 0.55)	0.16 (0.16, 0.16)	0.51 (0.50, 0.52)	1.78 (1.74, 1.82)	2.74 (2.69, 2.79)

Prevalence of diagnosed ET; per 1,000 patients (95% CI)	5.48 (5.43, 5.53)	1.59 (1.55, 1.64)	5.09 (5.02, 5.16)	17.83 (17.42, 18.23)	27.39 (26.87, 27.92)

^a^Individuals at risk include adults who are observable on each day of 2022 with at least 365 days of baseline observability prior to the start of 2022.

^b^Primary analysis: patients are considered to have diagnosed ET upon the second of at least two claims with a diagnosis of ET between 1 and 365 days apart during the study period.

^c^Sensitivity analysis 1: patients are considered to have diagnosed ET upon the second of at least two claims with a diagnosis of ET between 1 and 730 days apart during the study period.

^d^Sensitivity analysis 2: patients are considered to have diagnosed ET upon the occurrence of at least one claim with a diagnosis of ET during the study period.

Age assessed on 1/1/2022.

95% CI estimated as follows: letting n be the number of patients and p the observed proportion of successes, if np(1-p) < 5, the Clopper-Pearson method is used; otherwise, the Wald interval is used.

The study period starts on 1/1/2016 and extends through 12/31/2022.

CI, confidence interval; ET, essential tremor.

### 3.2. Proportion of adults with diagnosed ET who receive treatment commonly used to treat ET

Among 19,511 adults in the Diagnosed ET-Case Denominator Cohort (those with prevalent diagnosed ET according to the Primary ET-Case Definition who additionally had at least six months of enrollment in 2022 after first meeting the ET definition), 14,397 (73.8% [95% CI: 73.2%, 74.4%]) received at least one possible treatment for ET on or after their first diagnosis across all age groups. The proportion (95% CI) treated was numerically lowest among the youngest age group (52.5% [50.1%, 54.8%] treated among those age 18–40 years), followed by the 41–64 age group (73.1% [72.2%, 74.0%] treated), and numerically highest among the 65–74 and ≥75 age groups (78.7% [77.3%, 80.1%] and 78.5% [77.3%, 79.6%] treated, respectively) (Table 2).

**Table 2:** Proportion of patients receiving possible treatment in 2022 in MarketScan among patients with diagnosed essential tremor (ET), overall and by age subgroup.


	OVERALL: AGE ≥18 YEARS *NOT AGE-STANDARDIZED*	AGE SUBGROUPS			

		AGE 18–40 YEARS	AGE 41–64 YEARS	AGE 65–74 YEARS	AGE 75+ YEARS

Patients with diagnosed ET in 2022^a^; N	19,511	1,702	8,927	3,473	5,409

Patients diagnosed with ET who received possible treatment in 2022^b^; N	14,397	893	6,526	2,733	4,245

Proportion of patients who received possible treatment; % (95% CI)	73.79% (73.17%, 74.41%)	52.47% (50.10%, 54.84%)	73.10% (72.18%, 74.02%)	78.69% (77.33%, 80.06%)	78.48% (77.34%, 79.58%)

^a^Patients at risk include patients with at least two claims with a diagnosis of ET who are observable on each day of 2022, who have at least 365 days of baseline observability prior to the start of 2022, and 6 months (180 days) of additional follow-up observability following first ET diagnosis (second of two claims with a diagnosis code for ET, 1–365 days apart).

^b^Patients are considered to have received treatment if a claim for possible ET treatment is observed on or after the first ET diagnosis (second of two claims with a diagnosis code for ET, 1–365 days apart) during the study period.

Age assessed on 1/1/2022.

95% CI estimated as follows: letting n be the number of patients and p the observed proportion of successes, if np(1-p) < 5, the Clopper-Pearson method is used; otherwise, the Wald interval is used.

The study period starts on 1/1/2016 and extends through 12/31/2022.

CI, confidence interval; ET, essential tremor.

### 3.3. Treatment characteristics among adults with diagnosed ET who received possible treatment

Of the 14,397 adults with diagnosed ET who received possible treatments, beta blockers and anticonvulsants were the most common classes of possible medication for ET dispensed in 2022, with 65.3% of treated adults having at least one claim for a beta blocker and 53.6% having at least one claim for an anticonvulsant. The most common beta blockers dispensed were propranolol (42.0% with a claim, the most common ET medication observed overall) and metoprolol (20.7%); the most common anticonvulsants dispensed were primidone (26.6%) and gabapentin (22.8%). Benzodiazepines and antidepressants were dispensed to 17.7% and 14.1% of adults, respectively, with alprazolam being the most commonly observed benzodiazepine and trazodone being the most common antidepressant. Other ET medication classes were dispensed to <4% of adults with diagnosed ET who received possible treatment. Beta blocker use increased moderately by age group (ranging from 62.0% to 67.8% with a claim among the youngest and oldest age groups, respectively), while anticonvulsant use was numerically lowest among the youngest age group (39.9% with a claim) and highest among those age 65–74 years (57.8%). Benzodiazepine use was numerically lowest in those aged ≥75 years (12.7%). Use of surgical treatments among this population was low, with just 0.3% of adults receiving deep brain stimulation, 0.2% receiving radio-surgical gamma knife thalamotomy, and 0.1% receiving focused ultrasound in 2022 (Table 3).

**Table 3:** Top treatments by class and medication observed in 2022 in MarketScan following first observed prevalent essential tremor (ET) diagnosis, among adults with prevalent diagnosed ET who received possible treatment, overall and by age subgroup.


	OVERALL: AGE ≥18 YEARS *NOT AGE-STANDARDIZED*	AGE SUBGROUPS			

		AGE 18–40 YEARS	AGE 41–64 YEARS	AGE 65–74 YEARS	AGE 75+ YEARS

Patients with prevalent 2022 ET who received possible treatment^a^; N	14,397	983	6,526	2,733	4,245

**Patients with treatment in 2022 by treatment class; N (%)**					

Beta blockers	9,398 (65.3)	554 (62.0)	4,194 (64.3)	1,770 (64.8)	2,880 (67.8)

Anticonvulsants	7,723 (53.6)	356 (39.9)	3,414 (52.3)	1,581 (57.8)	2,372 (55.9)

Benzodiazepines	2,545 (17.7)	169 (18.9)	1,368 (21.0)	470 (17.2)	538 (12.7)

Antidepressants	2,037 (14.1)	134 (15.0)	902 (13.8)	415 (15.2)	586 (13.8)

Botulinum toxins (A)	499 (3.5)	42 (4.7)	297 (4.6)	73 (2.7)	87 (2.0)

Calcium channel blockers	428 (3.0)	10 (1.1)	152 (2.3)	104 (3.8)	162 (3.8)

Antipsychotics	196 (1.4)	28 (3.1)	82 (1.3)	37 (1.4)	49 (1.2)

Carbonic anhydrase inhibitors	78 (0.5)	13 (1.5)	31 (0.5)	9 (0.3)	25 (0.6)

Potassium channel blockers	6 (0.0)	0 (0.0)	5 (0.1)	1 (0.0)	0 (0.0)

**Patients with treatment in 2022 by medication; N (%)**					

Beta blockers: propranolol	6,048 (42.0)	502 (56.2)	3,104 (47.6)	1,014 (37.1)	1,428 (33.6)

Anticonvulsants: primidone	3,833 (26.6)	111 (12.4)	1,492 (22.9)	867 (31.7)	1,363 (32.1)

Anticonvulsants: gabapentin	3,280 (22.8)	127 (14.2)	1,524 (23.4)	655 (24.0)	974 (22.9)

Beta blockers: metoprolol	2,983 (20.7)	42 (4.7)	946 (14.5)	684 (25.0)	1,311 (30.9)

Antidepressants: trazodone	1,537 (10.7)	107 (12.0)	751 (11.5)	316 (11.6)	363 (8.6)

Benzodiazepines: alprazolam	1,402 (9.7)	80 (9.0)	721 (11.0)	270 (9.9)	331 (7.8)

Anticonvulsants: topiramate	1,371 (9.5)	95 (10.6)	710 (10.9)	266 (9.7)	300 (7.1)

Benzodiazepines: clonazepam	1,221 (8.5)	98 (11.0)	686 (10.5)	214 (7.8)	223 (5.3)

Anticonvulsants: pregabalin	761 (5.3)	31 (3.5)	391 (6.0)	174 (6.4)	165 (3.9)

Antidepressants: mirtazapine	589 (4.1)	38 (4.3)	183 (2.8)	117 (4.3)	251 (5.9)

Beta blockers: atenolol	489 (3.4)	14 (1.6)	202 (3.1)	102 (3.7)	171 (4.0)

Botulinum toxins: onabotulinumtoxinA	451 (3.1)	40 (4.5)	268 (4.1)	64 (2.3)	79 (1.9)

Anticonvulsants: levetiracetam	411 (2.9)	52 (5.8)	165 (2.5)	65 (2.4)	129 (3.0)

Calcium channel blockers: nifedipine	239 (1.7)	4 (0.4)	69 (1.1)	55 (2.0)	111 (2.6)

Antipsychotics: olanzapine	183 (1.3)	24 (2.7)	79 (1.2)	34 (1.2)	46 (1.1)

Calcium channel blockers: verapamil	190 (1.3)	6 (0.7)	84 (1.3)	49 (1.8)	51 (1.2)

Anticonvulsants: zonisamide	159 (1.1)	14 (1.6)	78 (1.2)	25 (0.9)	42 (1.0)

Beta blockers: nadolol	126 (0.9)	4 (0.4)	57 (0.9)	26 (1.0)	39 (0.9)

Beta blockers: sotalol	74 (0.5)	1 (0.1)	14 (0.2)	13 (0.5)	46 (1.1)

Carbonic anhydrase inhibitors: acetazolamide	61 (0.4)	13 (1.5)	27 (0.4)	4 (0.1)	17 (0.4)

Botulinum toxins: incobotulinumtoxinA	42 (0.3)	2 (0.2)	25 (0.4)	7 (0.3)	8 (0.2)

Antipsychotics: clozapine	13 (0.1)	4 (0.4)	3 (0.0)	3 (0.1)	3 (0.1)

Botulinum toxins: abobotulinumtoxinA	11 (0.1)	0 (0.0)	8 (0.1)	2 (0.1)	1 (0.0)

Carbonic anhydrase inhibitors: methazolamide	18 (0.1)	1 (0.1)	4 (0.1)	5 (0.2)	8 (0.2)

Anticonvulsants: perampanel	6 (0.0)	0 (0.0)	5 (0.1)	1 (0.0)	0 (0.0)

Beta blockers: pindolol	6 (0.0)	0 (0.0)	3 (0.0)	1 (0.0)	2 (0.0)

Calcium channel blockers: nimodipine	0 (0.0)	0 (0.0)	0 (0.0)	0 (0.0)	0 (0.0)

Potassium channel blockers: perampanel	6 (0.0)	0 (0.0)	5 (0.1)	1 (0.0)	0 (0.0)

**Patients treated with surgical therapies in 2022; N (%)**					

Deep brain stimulation	38 (0.3)	2 (0.2)	18 (0.3)	14 (0.5)	4 (0.1)

Radio-surgical gamma knife thalamotomy	24 (0.2)	0 (0.0)	6 (0.1)	10 (0.4)	8 (0.2)

Focused ultrasound	17 (0.1)	0 (0.0)	7 (0.1)	3 (0.1)	7 (0.2)

^a^Patients are considered to have received treatment if a claim for possible ET treatment is observed on or after the first ET diagnosis (second of two claims with a diagnosis code for ET, 1–365 days apart) during the study period. Age assessed on 1/1/2022. The study period starts on 1/1/2016 and extends through 12/31/2022. ET, essential tremor.

### 3.4. Projections to the US population after age standardization

After age-standardizing prevalence estimates to the 2024 US population, the estimated prevalence of diagnosed ET among adults in the US using the Primary ET-Case Definition (at least two ET-related claims within one year) was 0.42%, corresponding with 1,123,500 adults with diagnosed ET in the US in 2024. An estimated 851,590 adults in the US with diagnosed ET received possible treatment for ET in the last year (Table 4).

**Table 4:** Estimated number of patients with essential tremor (ET) in the United States (US) in 2024, adults overall and by age subgroup.


	OVERALL: ≥18 YEARS *AGE-STANDARDIZED ESTIMATE^a^*	AGE SUBGROUPS			

		18–40 YEARS	41–64 YEARS	65–74 YEARS	75+ YEARS

Number of people in the US population, 2024	267,400,939	104,825,019	99,221,580	35,876,763	27,477,577

**Primary analysis: At least two claims, 1–365 days apart** ^b^					

Prevalence of diagnosed ET (%)	0.42	0.06	0.25	0.97	1.61

Estimated number of patients in the US with diagnosed ET^c^	1,123,500	67,298	247,424	639,545	752,730

Proportion of patients with diagnosed ET who received possible treatment (%)	73.79	52.47	73.10	78.69	78.48

Estimated number of patients in the US with ET who received possible treatment^d^	851,590	35,310	180,877	273,542	348,120

**Sensitivity analysis 1: At least two claims, 1–730 days apart** ^e^					

Prevalence of diagnosed ET (%)	0.45	0.07	0.27	1.04	1.73

Estimated number of patients in the US with diagnosed ET^c^	1,210,726	73,871	269,581	374,156	474,477

**Sensitivity analysis 2: At least one claim** ^f^					

Prevalence of diagnosed ET (%)	0.78	0.16	0.51	1.78	2.74

Estimated number of patients in the US with diagnosed ET^c^	2,089,908	166,859	504,693	639,545	752,730

^a^The total number of adults age ≥18 years in the US diagnosed with ET is calculated by summing across the age-specific estimates. The age-specific N’s are calculated by applying the age group-specific adult prevalence estimates to the corresponding US population estimate.

^b^Primary analysis: patients are considered to have diagnosed ET upon the second of at least two claims with a diagnosis of ET between 1 and 365 days apart during the study period.

^c^(Prevalence of diagnosed ET [95% CI]) ×(Number of patients in the US population).

^d^(Proportion of patients with diagnosed ET at risk who received possible treatment) × (Number of patients in the US population).

^e^Sensitivity analysis 1: patients are considered to have diagnosed ET upon the second of at least two claims with a diagnosis of ET between 1 and 730 days apart during the study period.

^f^Sensitivity analysis 2: patients are considered to have diagnosed ET upon the occurrence of at least one claim with a diagnosis of ET during the study period.

Prevalence of diagnosed ET is calculated as described in Table 1. Proportion of patients with diagnosed ET is calculated as described in Table 2.

US population estimates are based on 2024 estimates from the International Database of the U.S. Census Bureau International Programs Center https://www.census.gov/data-tools/demo/idb/#/pop?menu=popViz.

ET, essential tremor.

Using an expanded definition allowing up to two years between ET claims, the estimated prevalence increased slightly to 0.45%, corresponding with 1,210,726 US adults with diagnosed ET. Expanding the case definition to require only one ET-related claim, the estimated US prevalence was 0.78%, corresponding with 2,089,908 US adults with diagnosed ET (Table 4).

## 4. Discussion

There were an estimated 1.1 million adults with diagnosed ET in the US in 2024, corresponding with an overall estimated prevalence of diagnosed ET among US adults of 0.42%, after age standardization. The prevalence of diagnosed ET numerically increased with age, which is consistent with available literature [11,13,18,19]. Of those with diagnosed ET, approximately three quarters received possible treatment for ET within 2022, with propranolol being the most common medication dispensed, followed by primidone, gabapentin, and metoprolol. Varying the case definition of ET to assess the robustness of the estimates impacted the estimated prevalence. Specifically, requiring only a single ET-related claim rather than at least two claims nearly doubled the prevalence estimate, while loosening restrictions on the allowed time between two required claims had only a modest impact. These findings demonstrate the importance of algorithm selection on the estimated prevalence of chronic conditions in real-world data sources. There is a need for a validated algorithm for ascertainment of ET cases in administrative claims databases to further support study of individuals with ET in real-world data.

A study using Compile insurance claims data projected 2.2 million adults in the US to have diagnosed ET as of 2019, evaluated over a five-year study period and defining ET as the presence of at least one claim for ET [20]. This definition is consistent with the most relaxed secondary definition evaluated in these analyses, which yielded an estimate of 2.1 million adults in the US with diagnosed ET in 2024. Using a more conservative case definition additionally requiring two presumptive prescriptions for ET or a diagnosis code for an unspecified tremor, the Compile study estimated approximately 770,000 cases of treated or otherwise confirmed ET, which is slightly lower than the estimated 840,000 individuals with diagnosed ET who were prescribed different medication classes with possible efficacy for ET in the primary analysis of the present study. These estimates may vary because of differences in underlying case definitions such as the number of required prescription claims, where the present study required only one prescription claim after meeting ET diagnostic criteria in order to satisfy treatment criteria. Additionally, the methods used to project to the US population differed. The current study used age-standardized projection with US census data to account for age differences in the MarketScan data, and the previous study assumed a database capture rate of 60% of the total US population; therefore, the diagnosis data were projected to 100%.

Importantly, the present study identified ET as diagnosed and cared for in a real-world setting, thus estimating the prevalence of clinically diagnosed ET but not capturing sub-clinical ET, nor ET not requiring clinical care. A 2014 meta-analysis of three age-standardized studies from Turkey, Israel, and Spain where ET was identified via neurological examination estimated the prevalence of ET in the US as 2.2% [11]. Additionally, a targeted literature review estimated that 6.4 million adults in the US have ET, with an overall prevalence of 2.6% [35]. The ET prevalence estimates from both of these studies were considerably higher than the age-standardized prevalence of diagnosed ET estimated in this study of 0.42%. Estimates of ET prevalence as identified via clinical evaluations and population-based studies are generally expected to be higher than the prevalence of ET identified in real-world claims-based analyses. This claims-based analysis aims to estimate the real-world prevalence of diagnosed ET, while population-based studies will also capture patients who have milder symptoms, may not seek medical attention, and who therefore will not be captured in claims-based data in real-world situations.

These analyses are subject to a number of limitations. While the G25.0 code is used to identify a diagnosis of ET in real-world settings [20], there is susceptibility to misclassification, despite a moderately high positive predictive value for ET (74.7%) [36]. More sensitive definitions (such as those requiring only one claim with a diagnosis of ET) may be more likely to capture false-positive cases due to the presence of a diagnosis code used for rule-out purposes. In this study, this potential misclassification was addressed by requiring at least two claims with a diagnosis for ET as the primary definition of ET. Conversely, ET is a slowly progressing condition that can go undiagnosed or very often be missed as a condition of interest when it is at a milder stage or not requiring active clinical management [6,12,20]. As such, increasingly specific case definitions may under-capture undiagnosed or well-managed ET, especially given limited longitudinality of claims data across an individual’s lifetime. In addition to the primary, more specific definition of ET, this study additionally evaluated more sensitive case definitions to address some of this potential under-capture. Given that the estimated prevalence of diagnosed ET nearly doubled with the requirement of only a single diagnosis compared with the primary case definition, this one-diagnosis definition may capture additional individuals with true ET who may be less clinically engaged (correct classification), as well as individuals without true ET, such as those with a rule-out diagnosis (misclassification).

A second limitation is that presumptive treatment for ET was defined as a prescription claim for medications with evidence indicating potential efficacious treatment of ET after meeting ET diagnostic criteria, but it is not known whether individuals actually took medications which were dispensed [37]. Further, many medications used for treatment of ET have additional indications, and a specific indication for the medication dispensed is not available in the administrative claims data; however, medications were only assessed among those with at least two medical claims related to ET [3,38]. Third, the prevalence of ET was evaluated among those with at least two years of continuous enrollment in a population of predominantly commercially insured adults under age 65 years; as a result, these estimates only represent those with a coded diagnosis who actively sought treatment at a physician office, hence generalizability to the overall adult US population may be limited. However, this study utilized age standardization to account for age differences in the MarketScan data versus the general US population to address this limitation, though residual differences in age distribution of the 2022 MarketScan data versus the 2024 US population within the age bands used for standardization may remain. It should be noted that diagnostic guidelines have not changed in recent years [34]; therefore, we do not expect that variation in diagnostic sensitivity between 2022 and 2024 would affect prevalence estimates. Future studies may explore the sensitivity of the G25.0 code and diagnostic patterns by provider types. Although it is standard to require 12 months of continuous enrollment after diagnosis in claims analyses, the 6-month requirement for this study’s analysis of possible ET treatment ensured that patients were properly captured, as the 12-month standard would have yielded artificially low estimates. Lastly, this study did not perform any statistical testing to measure increases in prevalence with age.

In conclusion, this study estimated 1.1 million individuals in the US in 2024 had diagnosed ET with a corresponding prevalence of 0.42%. Prevalence estimates numerically increased with age and were impacted by the changes in the case definition of ET, nearly doubling with the most sensitive definition used. Selection of an appropriate disease case definition is critical to estimating the prevalence of diagnosed ET in real-world data sources such as administrative claims, and utilization of methods such as standardization can be employed to address differences between the ET study sample and the general ET population of interest.

## Data Accessibility Statement

All relevant data are provided with the manuscript and supporting files.

## Additional File

The additional file for this article can be found as follows:

10.5334/tohm.1060.s1 Supplemental Materials. Supplemental Table 1.
