# Supplementary material for: Prevalence of Diagnosed Essential Tremor in the United States: An Administrative Claims-Based Study
Source: Tremor Other Hyperkinet Mov (N Y). 2025 Oct 21;15:51. doi: 10.5334/tohm.1060 (PMC12551629; doi:10.5334/tohm.1060)
Supplement: Supplemental Materials. — Supplemental Table 1. [file tohm-15-1-1060-s1.pdf]

TITLE: Prevalence of Diagnosed Essential Tremor in the United States: An Administrative Claims-Based Study

AUTHORS:
Junji Lin, PhD[1]; Rajesh Pahwa, MD[2]; Elan D. Louis, MD, MS[3]; Ragy Saad, MS[1,*]; Kelly E. Lyons, PhD[2]; Michael Markowitz, MD[1,*]; Liza R. Gibbs, MS[4]; Aisara Chansakul, MPH[4]; John Kroner, MS[4]; Douglas S. Fuller, MS[5]; Weiyi Ni, PhD[1]; Arthur Sillah, MPH, PhD[5]; Michelle Baladi, PhD[1,*]; Luigi M. Barbato, MD[5,*]; Sanket Shah, MBBS, PhD[5]

[1]Jazz Pharmaceuticals, Palo Alto, CA, USA; [2]University of Kansas Medical Center, Kansas City, KS, USA; [3]University of Texas Southwestern Medical Center, Dallas, TX, USA; [4]Aetion Inc, New York, NY, USA; [5]Jazz Pharmaceuticals, Philadelphia, PA, USA
*Former employee of Jazz Pharmaceuticals.

SUPPLEMENTAL MATERIALS

Supplemental Table 1. Pharmacologic Treatments With Evidence Indicating Potential Efficacy for Treatment of ET

Class	Medication	Generic name	CPT/HCPCS
Beta blockers	atenolol	ATENOLOL<br>ATENOLOL/CHLORTHALIDONE	
	metoprolol	METOPROLOL SUCCINATE<br>METOPROLOL SUCCINATE/HYDROCHLOROTHIAZIDE<br>METOPROLOL TARTRATE<br>METOPROLOL TARTRATE/DIETARY SUPPLEMENT,COMB.10<br>METOPROLOL TARTRATE/HYDROCHLOROTHIAZIDE	
	nadolol	NADOLOL<br>NADOLOL/BENDROFLUMETHIAZIDE	
	pindolol	PINDOLOL	
	propranolol	PROPRANOLOL HCL<br>PROPRANOLOL HCL/HYDROCHLOROTHIAZIDE	J1800 - INJECTION, PROPRANOLOL HCL, UP TO 1 MG
	sotalol	SOTALOL HCL	C9482 - INJECTION, SOTALOL HYDROCHLORIDE, 1 MG
Antidepressants	mirtazapine	MIRTAZAPINE	
	trazodone	TRAZODONE HCL<br>TRAZODONE HCL/DIETARY SUPPLEMENT NO.8	
Anticonvulsants	gabapentin	GABAPENTIN<br>GABAPENTIN ENACARBIL<br>GABAPENTIN/DIETARY SUPPLEMENT, MISC COMBO NO.11	
	levetiracetam	LEVETIRACETAM<br>LEVETIRACETAM IN SODIUM CHLORIDE, ISO-OSMOTIC	J1953 - INJECTION, LEVETIRACETAM, 10 MG
	perampanel	PERAMPANEL	
	pregabalin	PREGABALIN	
	primidone	PRIMIDONE	
	topiramate	TOPIRAMATE	
	zonisamide	ZONISAMIDE	
Benzodiazepines	alprazolam	ALPRAZOLAM<br>ALPRAZOLAM/DIETARY SUPPLEMENT,MISC	

		COMBO NO.17	
	clonazepam	CLONAZEPAM	
Antipsychotics	clozapine	CLOZAPINE	S0136 - CLOZAPINE, 25 MG
	olanzapine	OLANZAPINE<br>OLANZAPINE PAMOATE<br>OLANZAPINE/FLUOXETINE HCL	J2358 - INJECTION, OLANZAPINE, LONG-ACTING, 1 MG<br>S0166 - INJECTION, OLANZAPINE, 2.5 MG
Carbonic anhydrase inhibitors	acetazolamide	ACETAZOLAMIDE<br>ACETAZOLAMIDE SODIUM	J1120 - INJECTION, ACETAZOLAMIDE SODIUM, UP TO 500 MG
	methazolamide	METHAZOLAMIDE	
Botulinum toxins (A)	abobotulinumtoxinA	ABOBOTULINUMTOXINA	J0586 - INJECTION, ABOBOTULINUMTOXINA, 5 UNITS
	incobotulinumtoxinA	INCOBOTULINUMTOXINA	J0588 - INJECTION, INCOBOTULINUMTOXINA, 1 UNIT
	onabotulinumtoxinA	ONABOTULINUMTOXINA	J0585 - INJECTION, ONABOTULINUMTOXINA, 1 UNIT
Potassium channel blockers	perampanel	PERAMPANEL	
Calcium channel blockers	nifedipine	NIFEDIPINE<br>NIFEDIPINE, MICRONIZED	
	nimodipine	NIMODIPINE	
	verapamil	VERAPAMIL HCL	

CPT, Current Procedural Terminology; ET, essential tremor; HCPCS, Healthcare Common Procedure Coding System; HCL, hydrogen chloride; MISC, miscellaneous; MG, milligram.
